# Untangling population structure and genetic diversity of reticulocyte binding protein 2b (PvRBP2b) erythrocytic stage vaccine candidate in worldwide *Plasmodium vivax* isolates

**DOI:** 10.1371/journal.pone.0266067

**Published:** 2022-03-29

**Authors:** Leila Nourani, Akram Abouie Mehrizi, Sedigheh Zakeri, Navid Dinparast Djadid

**Affiliations:** Malaria and Vector Research Group (MVRG), Biotechnology Research Center (BRC), Pasteur Institute of Iran, Tehran, Iran; Fundação Oswaldo Cruz Centro de Pesquisas René Rachou: Fundacao Oswaldo Cruz Instituto Rene Rachou, BRAZIL

## Abstract

**Backgrounds:**

*Plasmodium vivax* is the predominant *Plasmodium* species distributed extensively in the Americas and Asia-Pacific areas. Encoded protein by *Plasmodium vivax* Reticulocyte Binding Proteins (PvRBPs) family member are of critical prominence to parasite invasion and have been considered the significant targets in development of malaria vaccine for the blood stage. As high genetic polymorphism of parasites may impede the effectiveness of vaccine development, more research to unraveling genetic polymorphism of *pvrbp2b* from various geographical regions seems indispensable to map the exact pattern of field isolates.

**Methodology/Principal findings:**

The aim of this study was to determine the sequences of Iranian *pvrbp2b* (nt: 502–1896) gene and then, to ascertain polymorphism of *pvrbp2b* gene, recombination, the level of genetic distances, evaluation of natural selection, and the prediction of B-cell epitopes of Iranian and global *P*. *vivax* isolates. *Pvrbp2b* partial gene was amplified and sequenced from 60 Iranian *P*. *vivax* isolates. Iranian *pvrbp2b* sequences as well as 95 published sequences from five countries were used to evaluate the genetic diversity and neutral evolution signature in worldwide scale. A total of 38 SNPs were identified among 60 Iranian *pvrbp2b* sequences (32 non-synonymous and 6 synonymous mutations), and 32 amino acid substitutions were observed in 29 positions as compared to Sal-1 sequence. Worldwide sequence analysis showed that 44 amino acid changes had occurred in 37 positions of which seven polymorphic sites had trimorphic mutations while the rest was dimorphic. The overall nucleotide diversity for Iranian isolates was 0.00431 ± 0.00091 while the level of nucleotide diversity was ranged from 0.00337 ± 0.00076 (Peru) to 0.00452 ± 0.00092 (Thailand) in global scale.

**Conclusions/Significance:**

Of amino acid substitutions, 12 replacements were located in the B-cell epitopes in which nine polymorphic sites were positioned in N-terminal and three polymorphic sites in predicted B-cell epitopes of C-terminal, signifying both variable and conserved epitopes for vaccine designing. Using the achieved outcome of the current investigation interrogate questions to the selection of conserved regions of *pvrbp2b* and understanding polymorphism and immune system pressure to pave a way for developing a vaccine based on PvRBP2b candidate antigen.

## Introduction

*Plasmodium vivax* is the predominant *Plasmodium* species distributed extensively in the Americas and Asia-Pacific areas [1], and 7.5–14.3 million cases of malaria mostly have been reported which nearly 56% belonging to South-Eastern part of Asia [2]. Regarding to *P*. *vivax*, relapses of the latent hypnozoites, high incidence of asymptomatic cases, the lack of continuous *in vitro* long-term culture system, occurrence of sever disease cases, multi-drug resistance strains and less vulnerability to frequently-used vector control procedures [3, 4], had drawn investigators’ attention to set the goal of eliminating malaria through arrangement and development of an effective vaccine [5]. The programs of malaria elimination and eradication have been initiated from 2007 with the objective of remarkable decline in malaria cases and mortality globally which eventually lead to the change of control stage to elimination of malaria in most endemic regions reported by WHO (2013). Thus, the identification of immunogenic and functional antigens which enable defensive immune responses is indispensable toward vaccine development. The induction of humoral immune responses by these *Plasmodium* antigens bring about complement intervention system through phagocytosis and/or direct killing of parasites to suppress merozoite invasion [6] and specific immune response by stimulating the release of cytokines which have a significant function against *P*. *vivax* parasite [7].

The capability of *P*. *vivax* to relapse from long-enduring dormant liver stages, asymptomatic blood-stage infections, shorter developmental process in *Anopheles* spp. body and the high level of genetic diversity, made the elimination of this parasite species more problematic than *P*. *falciparum* [8]. On the other hand, the greater level of genetic polymorphism in vaccine target antigens restricts specific immune responses to parasitic strains and decreases the subunit vaccine effectiveness [9]. The induced immune response against one haplotype may not stimulate responses to the other vaccine candidate haplotypes result in an elevation in the risk of the selection for non-vaccine strains in the vaccinated population [9]. To better assessment of immune response to each candidate antigen in the first step, understanding the genetic structure and the antigenic diversity of *P*. *vivax* populations, and in the next step, identification of the B-cell epitopes and the polymorphisms in immunogenic regions and detection of conserved epitopes, and defining regions undertaking neutral evolution in worldwide isolates are the key resolutions to overwhelm the matter.

Distinct clinical symptoms of malaria are resulted by blood stage infection. *P*. *vivax* merozoite invasion to the preferably reticulocytes is initiated by encoded protein of reticulocyte-binding protein gene family (PvRBPs) which are distributed on the different chromosomes (*pvrbp2c*, and *pvrbp2* (partial length) on chromosome 5; *pvrbp1a*, and *pvrbp1b* on chromosome 7; *pvrbp2b* on chromosome 8; *pvrbp2a*, *pvrbp2d*, *pvrbp2* (partial length), and *pvrbp3* on chromosome 14) [10]. PvRBP2b as 326 kDa protein, with a signal peptide at the N-terminus and putative transmembrane domain at the C-terminus, has a role in parasite invasion into reticulocytes for replication and transmission [11]. The analysis *pvrbp2b* showed that N-terminal is more polymorphic than C-terminal [10]. This efficacious and specific entry is facilitated by interactions between the invasion ligand, reticulocyte-binding protein 2b (*pvrbp2b*) and transferrin receptor 1 (TfR1) on the reticulocytes [12]. Cryogenic electron microscopy structure analysis revealed that the interaction of PvRBP2b and TfR1 is facilitated by its partial C-terminal domain which have a hidden surface area of approximately 1271 Å (located in residues 461 to 633), and Tf (Transferrin) via its N-terminal domain including a hidden external area of 386 Å (located in residues 168 to 460) [13]. Afterward, a sequence of molecular events are progressed that go along with preliminary bonding, recognition, attachment assurance and eventually diffusion of parasite into red blood cells [14]. Long-term cohort investigations in Brazil, Papua New Guinea (PNG) and Thailand revealed that PvRBP2b antibodies are associated with protection against *P*. *vivax* parasite by blocking complex construction between PvRBP2b and TfR1-Tf [15–17]. The preventive function of mouse monoclonal antibodies against PvRBP2b impede its binding to reticulocytes, in approximately 50% of clinical isolates from Brazil and Thailand [12]. Total IgG antibody levels of six member of PvRBPs family taken from individuals inhabiting in Brazil and Thailand, revealed that merely antibodies to PvRBP2b were related to protection against clinical malaria in both locations with low endemicity of *P*. *vivax* [16]. According to the antibody response in cohort studies, PvRBP2b (aa: 161–1454 and 1986–2653) showed the highest performance for categorizing new exposure and introduced as the top marker for detection of blood-stage *P*. *vivax* infections within the previous nine months in Thailand, Peru, and Brazil regions [18]. Furthermore, it has been shown that naturally acquired human antibodies against N-terminus of PvRBP2b can inhibit parasite invasion [19]. Therefore, due to the significant high polymorphisms [10] and potential to induce immune responses against PvRBP2b [12, 15–19], the N-terminal and a part of C-terminal region of *pvrbp2b* was considered for further analysis in the present study.

The malaria setting district Chabahar, Sistan and Baluchistan province, Iran is located neighboring Pakistan with an unstable and seasonal occurrence of malaria resulted by *P*. *falciparum* and *P*. *vivax* infections. During the last few years, a significant decrease in malaria cases has progressively happened in Iran from 3,108 to 1190 cases from 2011 to 2019 which more than 90% was due to the *P*. *vivax* infection [20]. The utilization of various interventions resulted in malaria cases decline over the malaria elimination program (Iranian CDMC, unpublished data, 2015). This success achieved by application of artemisinin combination therapy (ACT), as the first-line suggested approach for uncomplicated malaria caused by *P*. *falciparum*, larviciding, indoor residual spraying, long-lasting impregnated bed nets (LLINs), recognition of active cases and management of cases, enhanced diagnostic measurements like microscopy and RDT in different health services, reinforcing malaria control education system for involved individuals such as health workers, mobile groups in rural regions, and finally the amplified participation of community volunteers.

In the current study, primarily, the sequence of partial *pvrbp2b* gene was determined in Iranian *P*. *vivax* population and the polymorphism of *pvrbp2b* gene, recombination, the level of genetic distances, and the natural selection signature were investigated in Iranian isolates and compare them with other available global *pvrbp2b* sequences deposited in the GenBank or PlasmoDB databases. The second aim of the current investigation was to predict B-cell epitopes of PvRBP2b antigen in relation to amino acid replacements. The attained results would provide novel information associated to the antigenic diversity of PvRBP2b in global *P*. *vivax* isolates for designing and developing a PvRBP2b-based malaria vaccine.

## Materials and methods

### Study site, samples collection and *P*. *vivax* detection

This investigation used blood samples collected from 60 *P*. *vivax* infected patients with symptomatic malaria from an endemic area with low transmission rate in Sistan and Baluchistan province, located in south-east of Iran, from 2008 to 2012 [20]. Prior blood sampling, an informed consent was signed by adults or parents/legal guardians of children who contributed in the current experiment. To collect demographic and clinical information of each patient, a questionnaire was completed by the physician requiring data related to their age, nationality, gender, any exposure to malaria or travelling to highly transmitted areas, Afghanistan and Pakistan as neighbor malaria-endemic areas. An amount of 1 ml of peripheral blood collected from each individual, preserved into EDTA tubes which kept at -20°C until molecular analysis. The procedures executed in relation to human participants during the current investigation were approved by the ethical standards of the Institutional Review Board of Pasteur Institute of Iran. The majority of the blood donors were male (78.33%) which had a mean age of 25.8 years (range from 6 months to 70 years old) and were categorized by their nationality as Iranian (60%), Afghani (6.66%), and Pakistani (33.33%). *P*. *vivax* infection was approved via microscopic scrutinizing of blood smears and nested-PCR amplification of 18ssr DNA [21].

### Genomic DNA extraction, primer design and PCR assay

Parasite genomic DNA was extracted by using commercially available DNA purification kit (Promega, Madison, WI, USA) according to the manufacturer’s procedure according to the instructions provided by manufacturer and kept at -20°C until use. Oligonucleotide primers for amplification of *pvrbp2b* gene were designed in our laboratory according to Salvador I (Sal-I) *P*. *vivax* sequence, present in PlasmoDB (Accession number: PVX_094255). The amplification of *pvrbp2b* gene (partial, ~ 1700 bp) was performed by PCR using the oligonucleotide primers; external pairs F1 [5´-**AGC**AAACCTGAGAAGAAAACTACC-3´] and R1 [5´-ATCACGCTCGTGAAATGTATG-3´]. Underlined and bold nucleotides for *pvrbp2b* F1 primer indicate mismatches that were changed to adjust Tm and GC% of primers which showing the nucleotide differences with reference sequence (Sal-I *P*. *vivax*, PVX_094255). PCR reaction was performed in the final volume 25 μl including 0.5 pmol of each primer, 12.5 μl PCR master mix (AMPLIQON, Denmark), and 50 ng DNA template. The cycling condition for amplification was 95°C for 5 min, 25 cycles of 94°C for 1 min, annealing at 50°C for 1 min, and extending stage at 72°C for 1 min, followed by 72°C for 15 min. After amplification, all PCR products were analyzed by agarose gel electrophoresis 1% (Invitrogen, Carlsbad, CA, USA) and positive amplicons were purified and sequenced in both directions with external primers F1 and R1 and also with designed internal primers (FM1 [5´-AAAGGAAGCATTTAGGGGATG-3´] and RM1 [5´-ACTGTTAGCTGGTATGTTCTTTTG-3´]) by using Sanger sequencing procedure in GenFanavaran Group (Tehran, Iran). Nucleotide sequences were manually cleaned up, edited, aligned and then compared with the reference sequence (Sal-I, PVX_094255) by using ClustalW implemented in BioEdit v.7.1.7 [22] and MAFFT online version [23]. Nucleotide sequence data obtained in the current investigation were deposited in the GenBank database under the accession numbers OK416101 –OK416160. Translation of the sequences to amino acid sequences was performed by using Gene Runner software (v. 3.05, 1994; Hastings Software Inc.).

### Nucleotide diversity analysis in Iranian and global *pvrbp2b* sequences

The analysis of nucleotide diversity in Iranian *pvrbp2b* gene sequences (current study, n = 60) and the published sequences of *pvrbp2b* deposited in GenBank and PlasmoDB was performed using DnaSP v.5 [24]. Worldwide dataset comprising 155 *pvrbp2b* sequences from six different malaria endemic regions were analyzed in this study including Iran (current study, n = 60), Colombia (n = 22), Mexico (n = 10), Myanmar (n = 18), Peru (n = 28), and Thailand (n = 17). The number of haplotypes (H), haplotype diversity (Hd), segregating sites (S), total number of mutations (Eta), synonymous (Syn) and non-synonymous (NSyn), and nucleotide diversity (π) on a sliding window of 100 bp and a step size of 25 bp was estimated to measure the stepwise diversity of *pvrbp2b* gene. The number of singleton- (Si) and parsimonious-informative (Pi) sites were measured in MEGA6 [25]. The likelihood of the recombination between adjacent sites (R), the minimum number of recombination events (Rm) between different polymorphic sites, and average number of pair-wise nucleotide differences (K) were also calculated by using DnaSP package [24]. Natural selection’s effect for worldwide *P*. *vivax* isolates in *pvrbp2b* gene was used to estimate synonymous substitutions (dS) and non-synonymous substitutions (dN) per sites, according to the Nei and Gojobori’s method (1986) with Jukes and Cantor correction (1969). The positive dN-dS value designates to a positive selection. To evaluate the differences between dN and dS values, Z-test was performed by using MEGA6 software [25]. The other neutral analysis was assessed using calculation Tajima’s D, Fu and Li’s D* and Fu and Li’s F* values on a sliding window of 100 bp and a step size of 25 bp [26, 27]. In Tajima’s D, Fu and Li’s (D* and F*) analysis, positive value shows population decline or balancing selection or population structuring due to an excess of variants at intermediate frequencies. While, negative values is expected to be evidence of a population expansion or a directional selection due to the excess of rare variants (singleton sites) or low frequency alleles [26, 27].

The alignment of amino acid sequences was performed along with the reference sequence (Sal-I, PVX_094255) to define the substitutions across this antigen and the haplotypes were classified in global isolates. Haplotype network which represent the genetic distances among global haplotypes (n = 155 sequences), was constructed using Median-Joining method in PopART v1.7 [28] by DNA sequences. In order to avoid the recombination-generated biases, the input file were analyzed by RDP v.4.101 [29] and recombinant sites were removed. In this regard, the recombination events, major and minor parental isolates of recombinants, and recombination break points were analyzed in greater depth with different recombination detection methods implemented by in RDP v.4.101 program (with default configuration and Bonferroni corrected P-value 0.05) [29]. Moreover, to define genetic differences degree in worldwide *pvrbp2b* isolates, *F*_ST_ was estimated by DnaSP software [24].

### Prediction of B-cell epitopes in relation to SNPs

The prediction of linear B-cell epitopes of PvRBP2b and analysis to the presence or absence of polymorphisms in the predicted linear B-cell epitopes were accomplished using the ABCpred server (http://www.imtech.res.in/raghava/abcpred) under a threshold of 0.85. To envisage the conformational B-cell epitopes, the three-dimensional (3D) structure of PvRBP2b was retrieved from PDB site (Accession no. 6d03). Subsequently, conformational B-cell epitopes in PvRBP2b antigen was predicted using DiscoTope server version 2.0 (http://www.cbs.dtu.dk/services/DiscoTope) with 85% specificity. The anticipation of conformational B-cell epitopes in PvRBP2b as well as amino acid replacements in Iranian and worldwide isolates were mapped on the predicted 3D structure of PvRBP2b using WebLab ViewerLite v.4.2 (http://www.scalacs.org/TeacherResources) in a surface diagram.

## Results

### Genetic diversity and signatures of selection within Iranian and global *pvrbp2b* isolates

Amplification of *pvrbp2b* partial gene from 60 *P*. *vivax* isolates were resulted in ~1700 bp fragments on the agarose gel. All PCR products were successfully sequenced with external and internal primers. The genetic diversity analysis was performed on the fragments including N-terminal (nt: 502–1377) and C-terminal (nt: 1378–1896). A total of 38 SNPs were identified among 60 Iranian *pvrbp2b* sequences and the ratio of non-synonymous to synonymous substitutions was to 5.3:1 (32 non-synonymous and 6 synonymous mutations). The higher value of nucleotide diversity (π ± SD) was detected at N-terminal (0.00551 ± 0.00127) in comparison with C-terminal (0.00228± 0.00117) domain of Iranian *pvrbp2b* sequences. The higher rate of dN than dS was observed in N-terminal (*P* < 0.05, Z-test) but not in C-terminal domain of Iranian isolates (*P* > 0.05, Z-test). The non-significant negative value of Tajima’s D, Fu and Li (F* and D*) for each domain indicate the neutral evolution for Iranian *pvrbp2b* sequences (*P* > 0.05, Table 1).

**Table 1:** DNA sequence polymorphism analysis and neutrality test of Iranian *pvrbp2b* sequences in N-terminal (nt: 502–1377) and C-terminal (nt: 1378–1896) domains estimated by DnaSP and MEGA6 software (current study, n = 60).

Domains	S	Eta	H	Syn	NSyn	Si	Pi	Hd ± SD	K	π ± SD	Tajimas’ D	F* (F&L)	D* (F&L)	dS	dN	dN-dS	Z-test
**N-terminal**	27	29	33	5	24	11	16	0.963 ± 0.00013	4.829	0.00551 ± 0.00127	-0.7226	-1.7951	-1.9548	0.00220	0.00636	0.00400	**0.026**
**C-terminal**	8	9	11	1	8	1	7	0.684 ± 0.00411	1.184	0.00228± 0.00117	-1.0468	-0.4298	-0.0255	0.00151	0.00249	0.00090	0.303
**Total**	35	38	42	6	32	12	23	0.985 ± 0.00004	6.012	0.00431 ± 0.00091	-0.8672	-1.5689	-1.5774	0.00207	0.00491	0.00274	**0.038**

S: number of segregating sites, Eta: total number of mutations, H: haplotype, Syn: no. of synonymous mutations, NSyn: no. of non-synonymous mutations, Si: singleton sites, Pi: Parsimony informative sites, Hd: haplotype diversity, SD: Standard deviation, K: average number of pair-wise nucleotide differences, π: nucleotide diversity, D (Ti): Tajimas’ D value, dS: synonymous- and dN: non-synonymous nucleotide diversity (Pi(s), Jukes & Cantor), D*: Fu and Li’s D* test statistic, and F*: Fu and Li’s F* test statistic, *P* < 0.05 was considered significant for Z-test and were shown in bold numbers.

Nucleotide diversity (π) analysis of 155 isolates (nt = 502–1896) from six countries displayed 49 polymorphic sites which encompassed 5 synonymous and 44 non-synonymous sites. Nucleotide diversity estimation (π ± SD) for different geographical regions demonstrated the overall value of 0.00451 ± 0.000870 in worldwide *pvrbp2b* sequences. The highest nucleotide diversity was detected in Thailand (0.00452 ± 0.00092), while the lowest nucleotide diversity was calculated for Peru (0.00337 ± 0.00076) (Table 2).

**Table 2:** DNA polymorphism data and neutrality analysis for global *pvrbp2b* gene appraised by using DnaSP and MEGA6 programs (nt: 502–1896).

Country (n)	S	Eta	H	Syn	NSyn	Si	Pi	Hd ± SD	K	π ± SD	Rm (R)	D (Tj)	D* (F & L)	F* (F & L)	dS	dN	dN − dS	Z-test
**Colombia (22)**	14	14	9	3	11	2	12	0.8660 ± 0.051	5.17749	0.00371 ± 0.00074	2 (0.0084)	1.24477	0.75706	1.04932	0.00249	0.00404	0.0016	0.226
**Iran (60)** ^**a**^	35	38	42	6	32	12	23	0.9860± 0.006	6.01299	0.00431 ± 0.00091	7 (0.0342)	-0.86715	-1.57742	-1.56890	0.00207	0.00491	0.00274	**0.038**
**Mexico (10)**	14	14	4	2	12	0	14	0.7778 ± 0.091	5.95556	0.00427 ± 0.00095	0 (0.0008)	0.93172	1.51062 **	1.53943	0.00314	0.00459	0.0014	0.274
**Myanmar (18)**	19	19	7	1	18	6	13	0.6930 ± 0.114	4.77778	0.00342 ± 0.00091	2 (0.0011)	-0.52438	-0.04036	-0.20760	0.00131	0.00398	0.0026	**0.027**
**Peru (28)**	17	17	19	1	16	5	12	0.9600 ± 0.021	4.69577	0.00337 ± 0.00076	6 (0.0213)	0.25987	-0.16333	-0.03654	0.00149	0.00386	0.0024	**0.009**
**Thailand (17)** ^**b**^	19	20	13	1	19	5	14	0.9490 ± 0.044	6.30882	0.00452 ± 0.00092	4 (0.0313)	0.26286	0.32061	0.35161	0.00181	0.00524	0.0034	**0.030**
**Worldwide (155)**	46	49	85	5	44	10	36	0.9660 ± 0.009	6.29200	0.00451 ± 0.00087	10 (0.0262)	-0.97713	-1.40138	-1.46488	0.0021	0.0051	0.0030	**0.028**

S: number of segregating sites, Eta: total number of mutations, H: haplotype, Syn: no. of synonymous mutations, NSyn: no. of non-synonymous mutations, Si: singleton sites, Pi: Parsimony informative sites, Hd: haplotype diversity, SD: Standard deviation, K: average number of pair-wise nucleotide differences, π: nucleotide diversity, Rm: minimum number of recombination events, R: estimation of recombination between adjacent sites, D (Ti): Tajimas’ D value, dS: synonymous- and dN: non-synonymous nucleotide diversity (Pi(s), Jukes & Cantor), D*: Fu and Li’s D* test statistic (Statistical significance: **, *P* < 0.02)., F*: Fu and Li’s F* test statistic, *P* < 0.05 was considered significant for Z-test and were shown in bold numbers.

^a^ Current study

All global sequences retrieved from PlasmoDB database (http://plasmodb.org/plasmo/).

^b^ For Thailand, a total of 13 sequences were retrieved from PlasmoDB and four sequences were taken from NCBI [10].

**Fig 1:**
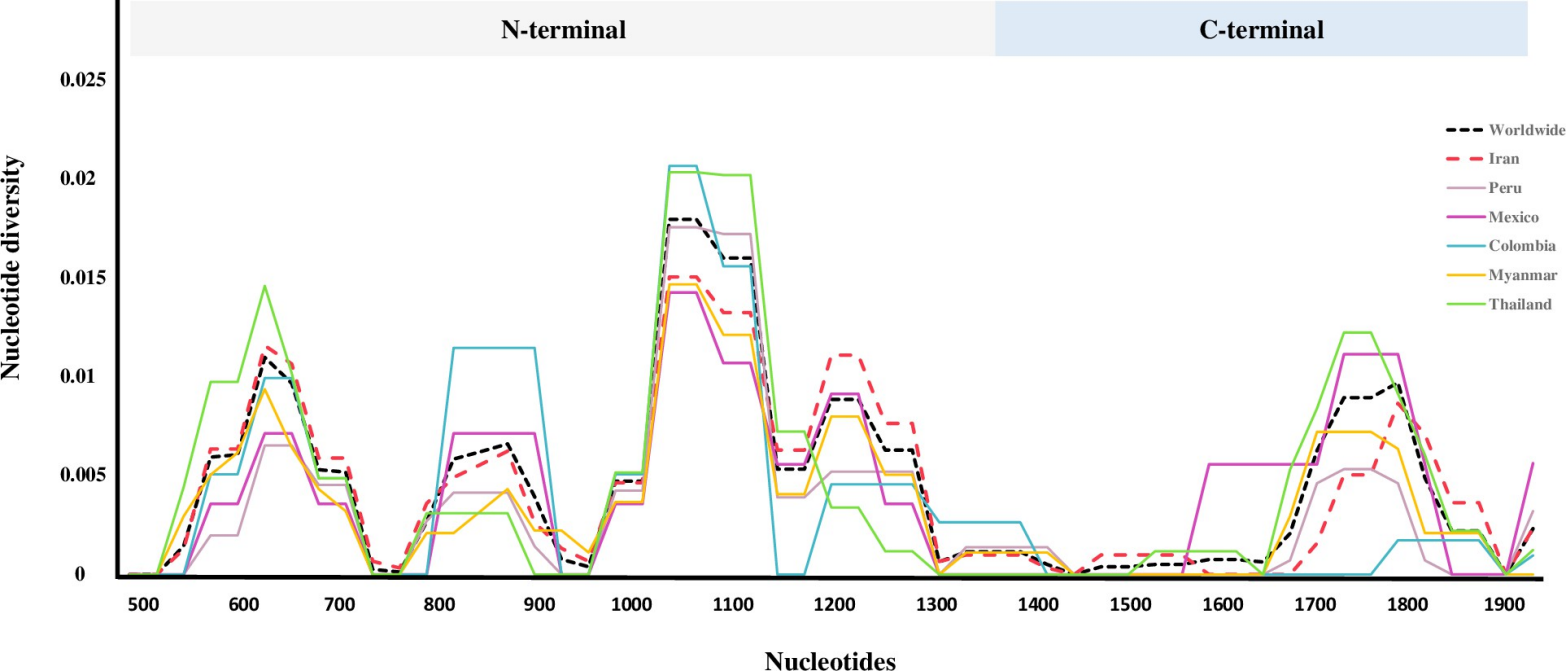
The plot represents the nucleotide diversity (π) across the global *pvrbp2b* DNA sequences (n = 155). The values demonstrates a window size of 100 bp with a step size of 25 bp which was calculated using DnaSP software. Nucleotides were numbered according to Sal-I reference sequence (PVX_094255).

The analysis of sliding window plot (window length of 100 bp and step size of 25 bp) revealed the nucleotide diversity values fluctuated from 0 to 0.02029 in worldwide *pvrbp2b* sequences. The slide window plot showed that the nucleotides with high diversity are in the N-terminal (Fig 1). Calculation of difference of dN-dS value on *pvrbp2b* gene was performed which demonstrated the significant positive value of dN-dS in the all studied countries (Z-test, *P* < 0.05), except for Colombia and Mexico (*P* > 0.05, Tables 1 and 2). Moreover, there was indication towards positive selection in worldwide scale, shown in Table 2 (dS < dN, P = 0.028).

**Fig 2:**
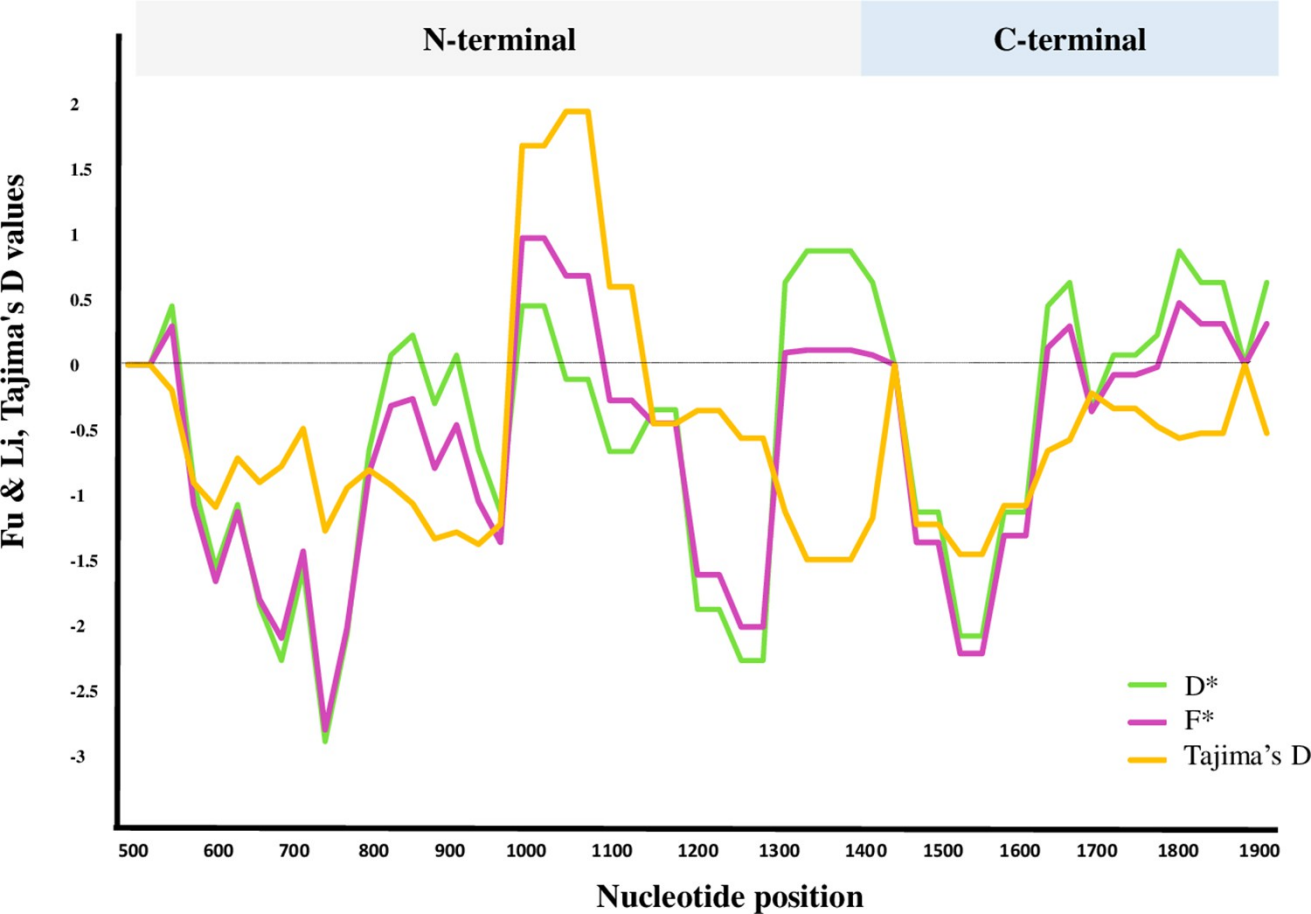
Tajima’s D, Fu and Li’s (D*& F*) values for global sequences of *pvrbp2b*. The values are measured on a sliding window of 100 bp and a step size of 25 bp to assess the stepwise diversity of worldwide dataset (*, *P* < 0.05). Nucleotides were numbered according to Sal-I reference sequence (PVX_094255).

**Fig 3:**
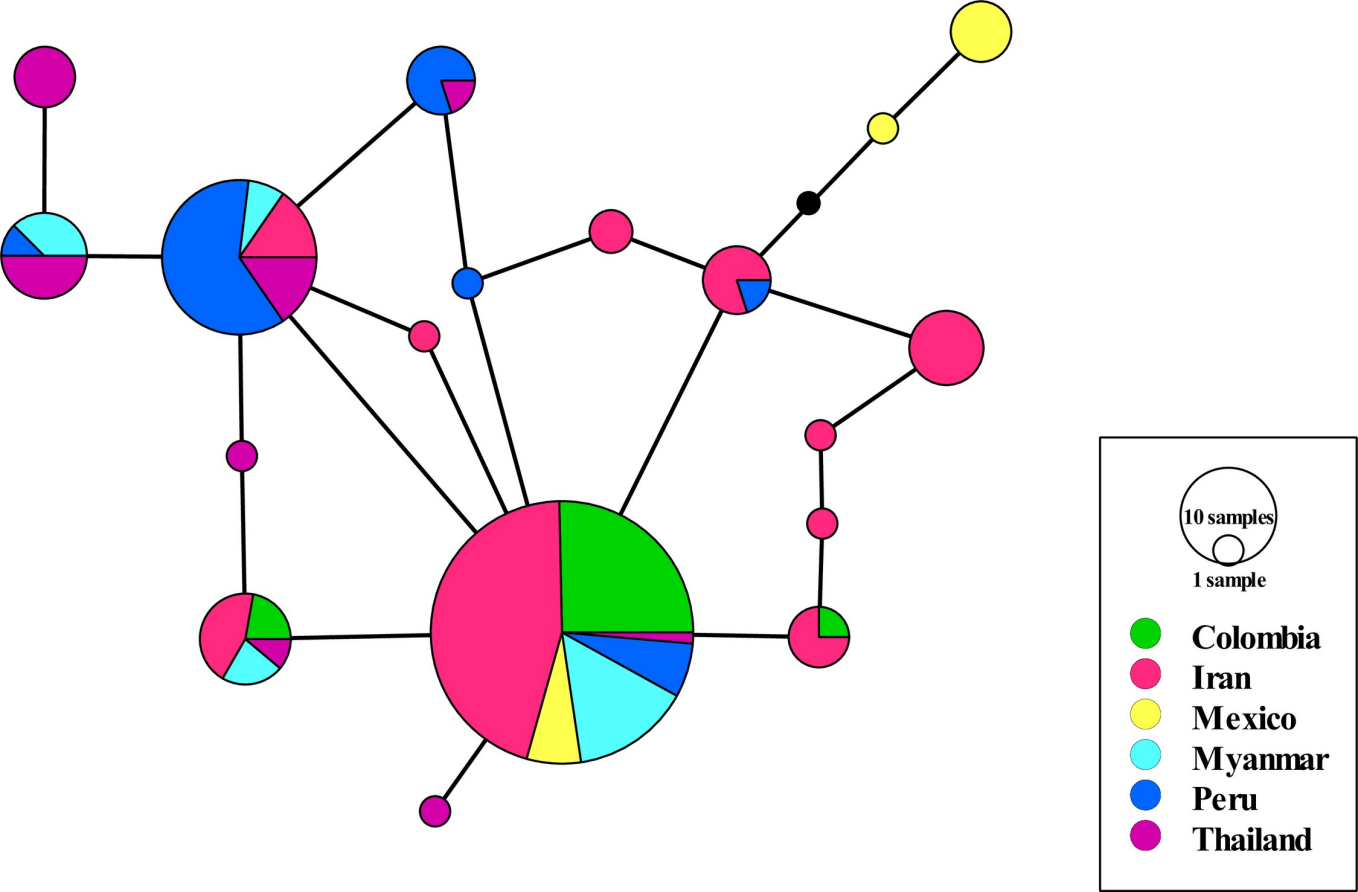
A haplotype network using *pvrbp2b* DNA sequences of global *P*. *vivax* isolates from Iran and other malaria endemic areas (n = 155). The median-joining method was used to construct the haplotype network in PopART software. The detected recombination sites were omitted before haplotype reconstruction by RDP v.4.101 software. The size of the circles specifies the haplotype frequency.

The analysis of neutrality selection for various geographical regions is shown in Table 2. The analysis showed non-significant negative values for Tajima’s D (-0.97713), Fu and Li’s D* (-1.40138), and Fu and Li’s F* (-1.46488) in *pvrbp2b* sequences in global scale (Fig 2, Table 2). The non-significant positive value of Tajima’s D revealed no departure from neutrality in Colombia, Mexico, Peru, and Thailand. Statistical calculation for D* values of worldwide sequences (n = 155) were only significant in window sliding sites 176–275, 226–325, and 701–825 (*P* < 0.05) and F* values were significant in window sliding sites 226-325(*P* < 0.05) (Fig 3). Tajima’s D not significant values calculated for worldwide sequences (n = 155) were consistent with Fu and Li (D* and F*) specify no departure from on neutral evolution theory (*P* > 0.05, Fig 2, Table 2).

Putative recombinants recovered by the RDP v.4.101 were confirmed by different methods. The results showed that five recombination events were found in which RDP and BootScan did not confirm significant values whereas one to four recombination events were affirmed by GENECONV, Chimaera, SiScan, MaXchi, and 3Seq methods (Table 3).

**Table 3:** Crossover sites in *pvrbp2b* sequences by using recombination detection methods in RDP v.4.101 program.

Event	Breakpoints		No. of Recombinant Sequence(s)	No. of Parental Sequence(s)		Methods						
	Begin	End		Minor	Major	RDP	GENECONV	BootScan	MaXchi	Chimaera	SiScan	3Seq
1	77	492	14	5	4	NS	NS	NS	4.79E-03	NS	NS	2.75E-03
2	105	514	21	5	1	NS	NS	NS	6.11E-03	4.84E-02	1.33E-02	1.54E-02
3	620	1024	1	6	19	NS	NS	NS	6.45E-03	NS	5.93E-03	NS
4	596	963	1	24	4	NS	NS	NS	NS	NS	NS	1.01E-02
5	424	619	86	3	1	NS	3.71E-02	NS	3.99E-03	NS	NS	1.01E-02

Minor and major parent = Parent contributing the smaller and larger fraction of sequence, respectively.

NS = No significant P-value.

### Haplotype diversity in worldwide sequences

Haplotype diversity was estimated based on variations in the worldwide *pvrbp2b* DNA sequences and the results demonstrated the presence of 85 haplotypes among 155 sequences. Different number of *pvrbp2b* haplotypes were found in Colombia (9 out of 22 isolates), Iran (42 out of 60 isolates), Mexico (4 out of 10 isolates), Myanmar (7 out of 18 isolates), Peru (19 out of 28 isolates), and Thailand (13 out of 17 isolates) (Table 2). Comparison of Iranian sequences with reference sequence (Sal-I *P*. *vivax*; PVX_094255) demonstrated 6.66% of haplotypes belong to the IRRBP2b-9 as predominant haplotype shown in Table 4.

**Table 4:** Haplotypes of Iranian *pvrbp2b* DNA sequences (current study, n = 60).

Nucleotide position (Sal-I) / Haplotypes (%)	G650A	G657T	G658T	C671A	G725C	G726C	A730C	G763C	A863C	A872C	T877G	T900A	A925C	C936T	G943T	T1047A	A1087G	A1089G	A1097T	C1098T	G1144A	T1184C	C1232A	A1236T/C	C1237A	A1338G	C1365A	A1479T	G1489A	A1691G/C	A1723G	C1758A	T1773A	A1878G	G1891A
**IRRBP2b-1 (5%)**	G	G	G	C	G	G	A	G	A	A	T	T	A	C	G	T	A	A	A	C	G	T	C	A	C	A	C	A	G	A	A	C	T	A	G
**IRRBP2b-2 (1.44%)**	.	.	.	.	.	C	.	.	C	.	.	.	.	.	.	.	.	.	.	.	.	.	.	.	.	.	.	.	A	.	.	.	A	.	.
**IRRBP2b-3 (1.44%)**	.	.	.	.	.	.	.	.	C	.	.	.	.	.	.	.	.	.	.	.	.	.	.	T	.	.	.	.	.	.	.	.	.	.	.
**IRRBP2b-4 (5%)**	.	.	.	.	.	.	.	.	.	.	.	.	.	.	.	.	.	.	.	.	.	.	.	T	.	.	.	.	.	.	.	.	.	.	.
**IRRBP2b-5 (5%)**	.	.	.	.	.	C	.	.	C	.	.	.	.	.	.	.	.	.	.	.	.	.	.	.	.	.	.	.	.	.	.	.	.	.	.
**IRRBP2b-6 (3.33%)**	.	.	T	.	.	.	.	.	.	.	.	.	.	.	.	A	G	.	T	T	.	.	.	T	.	.	.	.	.	.	.	.	.	G	.
**IRRBP2b-7 (1.44%)**	.	.	.	.	.	C	.	.	C	.	.	.	.	.	.	A	G	.	T	T	.	.	.	T	.	.	.	.	.	.	.	.	.	.	.
**IRRBP2b-8 (5%)**	.	.	.	.	.	C	.	.	.	.	.	.	.	.	.	A	G	.	.	.	A	C	.	.	A	.	.	.	.	.	G	A	.	.	.
**IRRBP2b-9 (6.66%)**	.	.	T	.	.	.	.	.	.	.	.	.	.	.	.	.	.	.	.	.	.	.	.	T	.	.	.	.	.	.	.	.	.	.	.
**IRRBP2b-10 (1.44%)**	.	.	.	.	.	C	.	.	.	.	.	.	.	.	.	A	G	.	.	.	A	.	.	.	.	.	.	.	.	.	G	A	.	.	.
**IRRBP2b-11 (1.44%)**	.	.	T	.	.	.	.	.	.	.	.	.	.	.	.	.	.	.	.	.	.	.	.	T	.	.	.	.	.	G	.	.	.	.	.
**IRRBP2b-12 (5%)**	.	.	T	.	.	.	.	.	.	.	.	.	.	.	.	.	.	.	.	.	.	.	.	.	.	.	.	.	.	.	.	.	.	.	.
**IRRBP2b-13 (1.44%)**	A	.	.	.	.	C	.	.	.	.	.	.	.	.	.	.	.	.	.	.	.	.	.	.	G	.	.	.	.	.	.	.	A	.	.
**IRRBP2b-14 (5%)**	.	.	.	.	.	C	.	.	C	.	.	.	.	.	.	.	.	.	.	.	.	.	.	.	.	.	.	.	.	G	.	.	.	.	.
**IRRBP2b-15 (1.44%)**	.	.	.	.	C	.	.	.	.	.	.	.	C	.	T	.	.	.	.	.	.	.	.	.	.	.	.	.	.	.	.	.	.	.	.
**IRRBP2b-16 (1.44%)**	.	.	T	.	.	.	.	.	.	.	.	.	.	.	.	.	.	.	.	.	.	.	.	.	.	.	.	.	.	.	G	.	.	.	.
**IRRBP2b-17 (1.44%)**	A	.	.	.	.	C	.	.	.	.	.	.	.	.	.	.	.	.	.	.	.	.	.	T	.	.	.	.	.	.	G	A	.	.	.
**IRRBP2b-18 (1.44%)**	.	.	T	.	.	.	.	.	.	.	.	.	.	.	.	.	.	.	.	.	.	C	.	T	.	.	.	.	.	C	.	.	.	.	.
**IRRBP2b-19 (3.33%)**	.	.	.	.	.	.	.	.	.	.	.	.	.	.	.	A	G	.	T	T	.	.	.	.	.	.	.	.	.	.	.	.	.	.	.
**IRRBP2b-20 (1.44%)**	.	.	T	.	.	.	.	.	.	.	.	.	.	.	.	A	.	.	.	.	.	.	.	T	.	.	.	.	.	.	.	.	.	.	.
**IRRBP2b-21 (1.44%)**	A	.	.	.	.	C	.	.	C	.	.	.	.	.	.	.	.	.	.	.	.	.	.	.	.	.	.	.	.	.	.	.	.	.	.
**IRRBP2b-22 (1.44%)**	.	.	.	.	.	.	.	.	.	.	.	.	.	.	.	A	.	G	.	.	A	C	.	.	A	.	.	.	.	.	G	A	.	.	.
**IRRBP2b-23 (1.44%)**	.	.	.	.	.	.	.	.	.	.	.	.	.	.	.	.	.	.	.	.	.	.	.	.	A	.	.	.	.	.	.	.	.	.	.
**IRRBP2b-24 (1.44%)**	.	.	.	.	.	C	.	.	.	.	.	.	C	.	.	A	G	.	T	T	.	.	.	.	.	.	.	.	.	.	.	.	.	.	.
**IRRBP2b-25 (1.44%)**	.	T	T	.	.	.	.	.	.	.	.	.	.	.	.	.	.	.	.	.	.	.	.	.	.	.	.	.	.	.	.	.	.	.	.
**IRRBP2b-26 (1.44%)**	.	.	T	.	.	.	.	.	.	.	.	.	.	.	.	A	G	.	T	T	.	.	.	C	.	.	.	.	.	.	.	.	.	.	.
**IRRBP2b-27 (3.33%)**	.	.	.	.	.	.	.	.	.	.	.	.	.	.	.	A	G	.	.	.	A	C	.	.	A	G	.	.	.	.	.	.	.	.	.
**IRRBP2b-28 (1.44%)**	.	.	T	.	.	.	.	.	.	.	.	.	.	.	.	.	.	.	.	.	.	C	.	.	.	.	.	.	.	.	G	.	.	.	A
**IRRBP2b-29 (1.44%)**	.	.	.	.	.	C	.	.	.	.	.	.	.	.	.	A	G	.	.	.	A	C	.	.	A	.	.	.	.	.	.	.	.	.	.
**IRRBP2b-30 (1.44%)**	.	.	.	.	.	C	C	C	.	.	G	A	.	T	.	A	G	.	T	T	.	.	.	.	.	.	.	.	.	.	.	.	.	.	.
**IRRBP2b-31 (1.44%)**	.	.	.	.	.	C	.	.	.	.	G	A	.	.	.	.	.	.	.	.	.	.	.	.	.	.	.	.	.	.	G	.	.	.	.
**IRRBP2b-32 (1.44%)**	.	.	.	.	.	C	.	.	.	.	.	.	.	.	.	A	G	.	.	.	A	C	.	.	A	.	A	.	A	.	.	.	.	.	.
**IRRBP2b-33 (1.44%)**	.	.	.	.	.	C	.	.	C	.	.	.	.	.	.	.	.	.	.	.	.	C	.	.	.	.	.	.	.	.	.	.	.	.	.
**IRRBP2b-34 (1.44%)**	.	.	.	.	.	.	.	.	.	.	.	.	.	.	.	A	G	.	T	T	.	.	.	T	.	.	.	.	.	.	.	.	.	G	.
**IRRBP2b-35 (1.44%)**	.	.	.	.	.	.	.	.	.	C	.	.	.	.	.	.	.	.	.	.	.	.	.	.	.	.	.	.	.	.	.	.	A	.	.
**IRRBP2b-36 (1.44%)**	.	.	.	A	.	.	.	.	.	.	.	.	.	.	.	.	.	.	.	.	.	.	.	.	.	.	.	.	.	.	.	.	.	.	.
**IRRBP2b-37 (1.44%)**	.	.	T	.	.	.	.	.	.	.	.	.	.	.	.	.	.	.	.	.	.	.	.	.	.	.	.	.	.	.	G	.	.	.	A
**IRRBP2b-38 (1.44%)**	.	.	.	.	.	C	.	.	C	.	.	.	.	.	.	.	.	.	.	.	.	.	.	.	.	.	.	.	.	.	G	.	.	.	.
**IRRBP2b-39 (1.44%)**	A	.	.	.	.	C	.	.	.	.	.	.	.	.	.	.	.	.	.	.	.	.	.	.	.	.	.	T	.	.	.	.	A	.	.
**IRRBP2b-40 (1.44%)**	.	.	.	.	.	C	.	.	.	.	.	.	.	.	.	A	G	.	.	.	A	C	A	.	.	.	.	.	.	.	.	A	.	G	.
**IRRBP2b-41 (1.44%)**	.	.	T	.	.	.	.	.	.	.	.	.	.	.	.	.	.	.	.	.	.	C	.	.	.	.	.	.	.	.	G	.	.	.	.
**IRRBP2b-42 (1.44%)**	.	.	T	.	.	.	.	.	.	.	.	.	.	.	.	A	G	.	T	T	.	.	.	T	.	.	.	.	.	.	G	A	.	G	.

To construction haplotype network, the detected recombination sites were omitted by RDP v.4.101 software to avoid crossing over sites bias. The number of detected haplotype after recombination sites omission, were 18 distinct haplotypes. Haplotype network analysis showed shared haplotypes for studied countries (Fig 3). Among detected haplotypes identified globally, the most frequent haplotype was found in Colombia, Iran, Mexico, Myanmar and Peru (~48%), whereas the second most prevalent haplotype was discovered among Iran, Myanmar, Peru and Thailand isolates (~17%).

### *F*_ST_ analysis

To analyze the population differences in *pvrbp2b* DNA sequences among malaria endemic regions, the *F*_ST_ values were calculated using *pvrbp2b* sequences from six countries of endemic malaria regions (n = 155). The positive *F*_ST_ value demonstrated genetic variations between populations which were observed in the all studied regions. High level of genetic distances (more than 0.25) was measured between Thailand and Mexico populations. Moderate level of genetic differentiation (0.1–0.25) was reported between Iran with Mexico, Peru and Thailand, and low level of genetic differentiation (0.01–0.09) was detected between Iranian population with Colombia and Myanmar. The results of *F*_ST_ analysis are summarized in Table 5. The highest and lowest value of genetic differences between Iranian isolates and other populations was observed in Thailand and Myanmar, respectively.

**Table 5:** Calculation of pairwise *F*_ST_ values for global *pvrbp2b* sequences (n = 155).

Countries	Colombia	Iran	Mexico	Myanmar	Peru	Thailand
**Colombia**		0.09367	0.19432	0.13999	0.17004	0.23040
**Iran**	0.0204 *		0.10504	0.05803	0.12420	0.24957
**Mexico**	0.0626	0.0799		0.14965	0.23450	0.34845
**Myanmar**	0.0546	0.0420 *	0.0358 *		0.05862	0.20176
**Peru**	0.0278 *	0.0298 *	0.0868	0.0783		0.13275
**Thailand**	0.0098 **	0.0216 *	0.0415 *	0.0289 *	0.1069	

*F*_ST_ values are shown in the upper right quadrant, and *P* values are shown in the lower left quadrant. *F*_ST_ is a measure of genetic distances between populations (*, 0.01 < *P* < 0.05; **, 0.001 < *P* < 0.01).

All global sequences retrieved from PlasmoDB database (http://plasmodb.org/plasmo/).

For Thailand, a total of 13 sequences were retrieved from PlasmoDB and four sequences were taken from NCBI [10].

### Polymorphic amino acid residues within global PvRBP2b

Appraisal of PvRBP2b sequences diversity with available dataset in six countries was executed by aligning amino acid residues with Sal-I reference sequence, and the results revealed 44 amino acid changes in 37 positions in the world sequences, of which seven polymorphic sites had trimorphic mutations while the rest was dimorphic. In Iranian isolates, 32 amino acid substitutions were observed in 29 positions of PvRBP2b antigen (Table 6). When comparing diversity across N- and C-terminal regions, amino acid changes were found to be more concentrated on N-terminal, shown in Table 1.

**Table 6:** The number of amino acid polymorphisms in different positions of PVRBP2b antigen, N-terminal (aa: 168–460) and C-terminal (aa: 461–633) among global isolates.

Amino acids position	Replaced aa	Colombia (n:22)	Iran (n:60)	Mexico (n:10)	Myanmar (n:18)	Peru (n:28)	Thailand (n:17)*	Worldwide (n:155)
**R217H**	H	-	4	-	3	-	5	12
**E219D**	D	-	1	-	-	-	-	1
**D220Y**	Y	9	19	2	-	3	1	34
**T224K/R**	K	-	1	-	-	-	2	3
	R	-	-	-	2	-	2	2
**E232K**	K	-	-	-	1	-	-	1
**R242S/T**	S	8	24	2	2	9	11	**56**
	T	-	1	-	1	-	-	2
**K244Q**	Q	-	1	-	-	-	-	1
**E255Q**	Q	-	1	-	-	-	-	1
**K288E/T**	E	-	-	-	2	3	3	8
	T	-	12	-	-	1	-	13
**K291T**	T	-	1	-	-	-	-	1
**L293V**	V	6	2	2	-	1	-	11
**N300K/D**	K	5	2	2	-	1	-	10
	D	4	-	-	-	-	-	4
**K309Q**	Q	-	2	-	1	-	-	3
**D315Y**	Y	-	1	-	1	-	-	2
**N349K**	K	13	21	2	4	8	10	**58**
**K363E**	E	13	19	2	4	9	11	**58**
**D366V**	V	11	10	2	4	9	10	**46**
**G382E/R**	E	-	-	-	1	7	4	12
	R	-	10	-	-	-	1	11
**V395A**	A	-	13	5	3	-	2	23
**P411Q**	Q	-	1	-	-	-	-	1
**K412N**	N	7	18	2	7	14	16	**64**
**Q413E/K**	E	-	1	-	-	-	-	1
	K	-	9	-	-	-	-	9
**E451K**	K	2	-	-	-	-	-	2
**H455Q**	Q	-	1	-	1	-	-	2
**N456Y**	Y	-	-	-	-	2	-	2
**L493F**	F	-	1	-	-	-	-	1
**E497K**	K	-	2	-	-	-	-	2
**T511A**	A	-	-	-	-	-	1	1
**V529M**	M	-	-	5	-	-	-	5
**D558E**	E	-	-	-	3	1	8	12
**S562I**	I	-	-	5	-	-	-	5
**Q564R/P**	R	-	4	-	5	21	14	44
	P	-	1	-	-	-	-	1
**P568S**	S	-	-	-	-	-	4	4
**K575E**	E	-	13	5	-	1	-	19
**S586R**	R	-	8	-	-	-	-	8
**N591K**	K	2	4	-	2	-	2	10
**E631K**	K	-	2	-	-	5	1	8

aa: amino acid

All global sequences retrieved from PlasmoDB database (http://plasmodb.org/plasmo/).

The shared amino acid replacements are in bold in worldwide column.

* For Thailand, a total of 13 sequences were retrieved from PlasmoDB and four sequences were taken from NCBI [10].

Region-specific amino acid replacements were identified in PvRBP2b sequences from Colombia (n = 2), Iran (n = 11), Myanmar (n = 1), Mexico (n = 2), Peru (n = 1), and Thailand (n = 2) (Table 6). The most frequent and shared replacements in different geographic regions were found at R242S, N349K, K363E, D366V, and K412N which were found in all countries.

### Analysis of the PvRBP2b structure in relation to antigenicity

**Fig 4:**
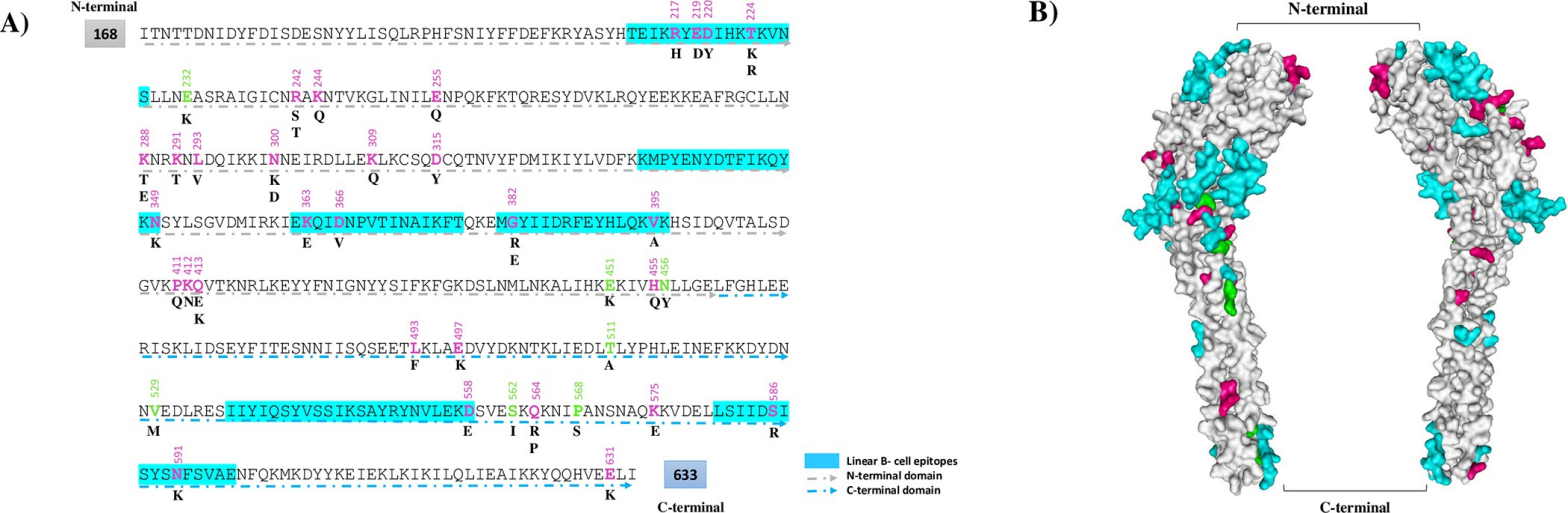
**A)** Amino acid sequence of PvRBP2b (Sal-I sequence, PVX_094255). The SNPs positions are specified in bold format and pink (detected SNPs in Iranian and worldwide populations), green color (detected SNPs in worldwide except Iran), and blue boxes show the predicted linear B-cell epitopes. The grey and blue arrows represent N- and C-terminal regions, respectively. **B)** The predicted conformational B-cell epitopes are mapped on 3D-structure of PvRBP2b (PDB code: 6d03) by using WebLab Viewer Lite 4.2. The color of the detected SNPs in Iranian population is shown in pink and detected SNPs in worldwide except Iran is green. In addition, the predicted conformational B-cell epitopes are in blue and white represents backbone amino acids.

The relationship between amino acid replacements and immunogenic regions of PvRBP2b antigen was evaluated via antigenic diversity analysis in B-cell epitopes. The linear B-cell epitopes prediction across the PvRBP2b antigen revealed that amino acids 213–228, 334–349, 362–377, 381–396, 536–558 and 581–596 were included in B-cell epitope regions, in which 12 polymorphic amino acids in Iranian isolates were found across the potential B-cell epitopes (Fig 4). Among polymorphic amino acid residues placing in linear B-cell epitope regions, R217H, E219D, D220Y, T224K, N349K, K363E, D366V, G389R, and V395A were located in N-terminal and the others (D558V, S586R, and N591K) were located in C-terminal (Fig 4). Furthermore, the prediction of conformational B-cell epitopes showed discontinuous regions involving in antibody binding (aa: 168–171, 173, 183–184, 213, 216–227, 252–253, 256–272, 294, 297–302, 304–305, 308–309, 313, 336, 338–349, 366, 378–369, 375, 379, 382, 386, 388–390, 393, 417, 441, 444–445, 448–449, 452, 479, 484, 515–516, 518–520, 522–523, 526, 566, 569–570, 572, 599, 602 and 627). Five amino acid replacements (N300K/D, K309Q, N349K, D366V, and G382E/R) are positioned in the predicted conformational B-cell epitopes. The predicted conformational B-cell epitopes and polymorphic residues in Iranian and global isolates were mapped on the retrieved 3D predicted structure of PvRBP2b antigen (Fig 4). Most of the conformational B-cell epitope residues were placed in N-terminal of PvRBP2b antigen.

## Discussion

Novel intervention tools and approaches like effective vaccine are essential to achieve malaria elimination and eradication. To address this goal, inspecting the dynamics of genetic diversity and defining polymorphism level in vaccine candidate antigens in various geographical regions, with varied endemicity of malaria, pave a way for designing and development of an efficient vaccine against *P*. *vivax* as the most distributed species outside Africa. This is the first investigation to estimate genetic diversity of *pvrbp2b* gene as well as SNPs which might be involved in potential B-cell epitopes of clinical *P*. *vivax* isolates from the tropical Southeastern area of Iran, a setting of low endemicity of malaria, and compare with worldwide populations.

Analysis of genetic diversity demonstrated a considerable level of nucleotide diversity (π ± SD, 0.00431 ± 0.00091) among Iranian *pvrbp2b* sequences. This result was similar to another investigation on erythrocytic antigens in Iran, region II of Duffy binding protein (0.00494 ± 0.00046) [30], but lower value than the diversity of Iranian sequences of apical membrane antigen 1 (0.00826 ± 0.00040) [31]. High percentage of nucleotide diversity in N-terminal (0.00551 ± 0.00127) versus C-terminal (0.00228 ± 0.00117) domain and haplotype frequency in this geographic area, signifies that N-terminal maybe under the pressure of immune system. Though, the high rate of polymorphism in erythrocytic antigens are considered as a strategy to escape from immune system evasion [32], due to the lower nucleotide diversity in *pvrbp2b* C-terminal domain, this part of protein can be regarded as a suitable vaccine candidate for further investigations. The analysis of nucleotide diversity in global scale showed that the lowest diversity in *pvrbp2b* isolates from Peru whereas the highest values belonged to Thailand population. Besides, nucleotide diversity for Iranian isolates was approximately similar to worldwide sequences. Due to the fact that nucleotide diversity degree is associated with the malaria transmission intensity in endemic regions [33], the greater values of diversity lead to the potential occurrence of polyclonal infections, varied gametocytes composition in *Anopheles* vectors, higher likelihood of recombination and creation of novel allelic forms [32]. The high value of the recombination parameters indicate that high meiotic recombination may occur between the sites, resulting in genetic diversity in the gene [34]. Investigation the recombination parameters of *pvrbp2b* gene showed the probability of development of new allelic forms in different countries, in which Peru and Iran showed the higher values of recombination after worldwide sequences.

Haplotype diversity demonstrated the high value in most of the countries included in the current study (worldwide; Hd ± SD: 0.9660 ± 0.0090). It was much more pronounced in Iranian isolates (Hd ± SD: 0.9860± 0.006) confirming the more variability (42 haplotypes out of 60 isolates) that may be due to new polymorphisms. In Myanmar, haplotype diversity was the least value in comparison with the other studied population (Hd ± SD: 0.6930 ± 0.114). Haplotype diversity and polymorphism are mostly related to various factors including recombination events, DNA mutation, marker determination and also demography [35]. Moreover, haplotype diversity reveal information about genetic diversity of parasites that are sampled [35]. It should be considered that Iranian blood samples are collected between the years that elimination program started in Iran (2010) and observations in the studied countries indicate the role of high transmission intensity and recombination in rising of nucleotide diversity and SNPs polymorphisms. Iran, Mexico, and Peru are low transmission areas while Thailand, Myanmar, and Colombia are high transmission regions. However, the singleton haplotypes were frequently found in both low and high transmission areas. Altogether, some factors such as sequencing inaccuracy, transmission intensity and prompt expansion of population also cause the greater occurrence of singleton haplotypes in parasitic isolates [36]. Respecting, the high frequency of haplotypes is a mechanisms to avoid immune system response, a multi-component vaccine depend on PvRBP2b antigen can be regarded to makes up for the entire immune-dominant areas in various haplotypes [35].

The distribution of polymorphic sites throughout PvRBP2b signify the more possibility of exposure with human immune responses and also highlights the significant immunogenicity of this antigen which was documented in recent investigation [16]. A total of 32 amino acid alterations were detected in PvRBP2b of Iranian isolates whereas global estimations showed 44 changes at amino acids positions. In PvRBP2b sequences, 12 amino acid replacements (T224R, E232K, K288E, N300D, G382E, E451K, N456Y, T511A, V529M, D558E, S562I, and P568S) were found in worldwide isolates except Iran which mostly located in N-terminal (Fig 4). It should be noted that the antigenic diversity is related to the malaria transmission intensity in different geographical regions [33]. However, this matter was not confirmed for the populations in the current study with different endemicity, as various populations showed almost the same diversity for both low and high endemic areas.

The higher frequency of dN rather than dS substitutions resulted in an increase grade of genetic diversity in a given gene [37]. In the current study, genetic diversity analysis showed that global *pvrbp2b* sequences had higher dN mutations than dS (2.42, *P* < 0.05) which specify positive selection. However, neutrality test using the Tajima’s D and Fu statistic did not confirm signature of positive selection in N- and C-terminal region of *Pvrbp2b* gene. It seems that negative values of Tajima’s D are due to the rare polymorphisms in these populations while dN>dS in several populations show positive selection. This contradiction in the results of tests may be due to low frequency polymorphisms and limited number of sample size leading to unreliable signal of Tajima’s D. The low level of synonymous mutations than non-synonymous polymorphism amongst *pvrbp2b* sequences is reported in other investigations. The dN/dS ratios for PvRBP2b family members have been reported as 3.0 (*pvrbp2a*), 3.1 (*pvrbp2b*), 2.4 and 3.9 (*pvrbp2c*) [10] and 4.5 (*pvrbp1*) [38]. In the current study, NS/S value was 2.37 for investigated Iranian isolates represent the lower value in comparison with the previous studies [10] and worldwide *pvrbp2b* sequences (2.42, shown in Table 2).

The significant pairwise *F*_ST_ values specify the high DNA divergence among studied populations. Our results revealed a high and a significant *F*_ST_ value between Iranian and other *P*. *vivax* populations. In the current study, significant pairwise *F*_ST_ values was reported for Iranian *pvrbp2b* sequences and Colombia, Peru, and Thailand (0.01 < *P* < 0.05). The extent and significant amount of pairwise *F*_ST_ confirm the absence of the gene flow due to geographical distance between populations and/or obstacle that can lead to the distinct genetic structure, as shown by the presence of distinct haplotypes in the haplotype network of *pvrbp2b*. This may be happen due to factors such as different sampling time, barriers that lead to lower movements, competency of *Anopheles* species, different environmental and climatic factors on mosquitoes and circulation of parasites [39]. The availability of similar SNPs (point mutations) among parasite populations may result in low *F*_ST_ value (close to zero) which have been observed in some genetic markers [40].

Isolation and characterization of anti-PvRBP2b human monoclonal antibodies (mAbs) in Cambodian patients proved the inhibitory role of naturally acquired antibodies against *P*. *vivax* invasion [19]. The majority of PvRBP2b human mAbs were connected to epitopes within amino acids 169–470, and 470–652, and only two mAbs were related to 969–1454 residues [19]. To know whether antigenic diversity and amino acid replacements can impact on the recognition of epitopes by antibodies, epitope prediction was performed and the results showed that of 44 substitutions, 14 replacements were located in the linear and conformational B-cell epitopes. Among predicted B-cell epitopes, 11 and three were positioned in polymorphic sites in N-terminal and C-terminal domains, respectively. This finding signifies the presence of immune system pressure in spite of existence of several conserved domains that can be considered in PvRBP2b-based vaccine design. The presence of some mutations in B-cell epitopes, implying that these mutations may alter the epitopes, leading to creation new epitopes that may be lead to unrecognition by the same antibodies. These mutations may hamper on the vaccine designed based on reference alleles. In this regard, in a recent study has been shown that the binding activity of a monoclonal antibody against PvRBP2b antigen may be influenced by K363 and G382 polymorphisms in N-terminal domain [19]. Interestingly these mutations were observed in Iranian and worldwide isolate (Table 6) and should be considered in vaccine design perhaps by including different variants to cover all mutations in these positions. Besides, Chan et al., declared that one (237235) out of four detected human antibody sets is located in a conserved region within the N-terminal domain. They found that this monoclonal antibody bind to amino acids 171, 173, 174, 193, 203, 231, 235, 238, 239, 241, 434, 437, 438, 441, 445 and 448 on the PvRBP2b antigen [19]. Interestingly, no polymorphism was detected in the mentioned positions of the analyzed worldwide sequences, which should be considered for PvRBP2b-based vaccine design. Moreover, C-terminal with fewer polymorphic sites is the target for potential human mAb [19] that may be considered as promising vaccine candidates.

## Conclusion

The current investigation analysis is in parallel to the restricted present data which demonstrated genetic diversity in the global *pvrbp2b* gene. These results indicate genetic diversity and geographic distances that could have an impact on the distribution of parasite strains. Several region-specific SNPs detected in Iranian and worldwide isolates, especially that mutation in B-cell epitopes as well the conserved regions might be considered in vaccine designing based on PvRBP2b antigen. These results are utilized for understanding the nature of the *P*. *vivax* population in Iran and other malaria-endemic regions, and are of remarkable importance for the development of PvRBP2b-based malaria vaccine.
