# Correction: Untangling population structure and genetic diversity of reticulocyte binding protein 2b (PvRBP2b) erythrocytic stage vaccine candidate in worldwide *Plasmodium vivax* isolates

**DOI:** 10.1371/journal.pone.0307447

**Published:** 2024-07-16

**Authors:** Leila Nourani, Akram Abouie Mehrizi, Sedigheh Zakeri, Navid Dinparast Djadid

## Notice of Republication

This article was republished on July 5, 2024, to correct the following errors. The publisher apologizes for these errors.

There is an error in the affiliation for all of the authors. The correct affiliation is: Malaria and Vector Research Group (MVRG), Biotechnology Research Center (BRC), Pasteur Institute of Iran, Tehran, Iran.

There is an error in the caption of Table 1, phrases “Syn: no. of synonymous mutations, NSyn: no. of non-synonymous mutations,” should be placed after the 3^rd^ comma, between haplotype and singleton sites. Please see the correct Table 1 here.

**Table 1:** 

Domains	S	Eta	H	Syn	NSyn	Si	Pi	Hd ± SD	K	π ± SD	Tajimas’ D	F* (F&L)	D* (F&L)	dS	dN	dN-dS	Z-test
**N-terminal**	27	29	33	5	24	11	16	0.963 ± 0.00013	4.829	0.00551 ± 0.00127	-0.7226	-1.7951	-1.9548	0.00220	0.00636	0.00400	**0.026**
**C-terminal**	8	9	11	1	8	1	7	0.684 ± 0.00411	1.184	0.00228± 0.00117	-1.0468	-0.4298	-0.0255	0.00151	0.00249	0.00090	0.303
**Total**	35	38	42	6	32	12	23	0.985 ± 0.00004	6.012	0.00431 ± 0.00091	-0.8672	-1.5689	-1.5774	0.00207	0.00491	0.00274	**0.038**

S: number of segregating sites, Eta: total number of mutations, H: haplotype, Syn: no. of synonymous mutations, NSyn: no. of non-synonymous mutations, Si: singleton sites, Pi: Parsimony informative sites, Hd: haplotype diversity, SD: Standard deviation, K: average number of pair-wise nucleotide differences, π: nucleotide diversity, D (Ti): Tajimas’ D value, dS: synonymous- and dN: non-synonymous nucleotide diversity (Pi(s), Jukes & Cantor), D*: Fu and Li’s D* test statistic, and F*: Fu and Li’s F* test statistic, *P* < 0.05 was considered significant for Z-test and were shown in bold numbers.

In the Genomic DNA extraction, primer design and PCR assay subsection of the Materials and Methods, there is an error in the third sentence of the first paragraph. The correct sentence is: The amplification of pvrbp2b gene (partial, ~ 1700 bp) was performed by PCR using the oligonucleotide primers; external pairs F1 [5´-**AGC**AAACCTGAGAAGAAAACTACC-3´] and R1 [5´-ATCACGCTCGTGAAATGTATG-3´]. There is also an error in the accession number at the end of the subsection. The correct accession number is: OK416101—OK416160.

Please download this article again to view the correct version. The originally published, uncorrected article and the republished, corrected article are provided here for reference.

## Supporting information

S1 File Originally published, uncorrected article (PDF)

S2 File Republished, corrected article (PDF)
